# A *Madurella mycetomatis* Grain Model in *Galleria mellonella* Larvae

**DOI:** 10.1371/journal.pntd.0003926

**Published:** 2015-07-14

**Authors:** Wendy Kloezen, Marilyn van Helvert-van Poppel, Ahmed H. Fahal, Wendy W. J. van de Sande

**Affiliations:** 1 Erasmus University Medical Center, Department of Medical Microbiology and Infectious Diseases, Rotterdam, The Netherlands; 2 St. Elisabeth Ziekenhuis, Department of Clinical Pathology, Tilburg, The Netherlands; 3 Mycetoma Research Centre, Soba University, Khartoum, Sudan; Fundação Oswaldo Cruz, BRAZIL

## Abstract

Eumycetoma is a chronic granulomatous subcutaneous infectious disease, endemic in tropical and subtropical regions and most commonly caused by the fungus *Madurella mycetomatis*. Interestingly, although grain formation is key in mycetoma, its formation process and its susceptibility towards antifungal agents are not well understood. This is because grain formation cannot be induced *in vitro*; a mammalian host is necessary to induce its formation. Until now, invertebrate hosts were never used to study grain formation in *M*. *mycetomatis*. In this study we determined if larvae of the greater wax moth *Galleria mellonella* could be used to induce grain formation when infected with *M*. *mycetomatis*. Three different *M*. *mycetomatis* strains were selected and three different inocula for each strain were used to infect *G*. *mellonella* larvae, ranging from 0.04 mg/larvae to 4 mg/larvae. Larvae were monitored for 10 days. It appeared that most larvae survived the lowest inoculum, but at the highest inoculum all larvae died within the 10 day observation period. At all inocula tested, grains were formed within 4 hours after infection. The grains produced in the larvae resembled those formed in human and in mammalian hosts. In conclusion, the *M*. *mycetomatis* grain model in *G*. *mellonella* larvae described here could serve as a useful model to study the grain formation and therapeutic responses towards antifungal agents in the future.

## Author Summary

Mycetoma is a chronic subcutaneous infectious disease affecting different parts of the body but commonly seen in the foot. It can be caused by bacteria and fungi. Especially for fungi, the treatment options are meagre and therapeutic failures are common. In order to develop better therapeutic strategies for this disease, models are needed which mimic the state of the causative agent inside the patient. Unlike other fungal pathogens, mycetoma causative agents produce a protective structure surrounding the hyphae. The hyphae embedded in this protective material are called a grain. This grain cannot be produced *in vitro*. A mammalian host is needed. In search for alternatives for animal use, larvae of the greater wax moth are often used as model systems for various infectious diseases. We therefore determined if these larvae were able to produce mycetoma grains. In this paper we describe the development of a *Madurella mycetomatis* grain model in *Galleria mellonella* larvae. We compare the grains formed in the larvae with those obtained from human patients and a previously developed mouse model.

## Introduction

Mycetoma is a chronic granulomatous subcutaneous infectious disease, characterized by massive deformities and disabilities. It is endemic in tropical and subtropical regions. It can be caused by 56 different micro-organisms, including both bacteria (actinomyctoma) and fungi (eumycetoma) [1]. The most common causative agent world-wide is the fungus *Madurella mycetomatis* [1]. A characteristic feature of mycetoma is the presence of grains inside the tissue. These grains are formed by the micro-organisms upon entering the human body, probably as a defense mechanism against the human immune system [2]. Since so many different micro-organisms are able to cause mycetoma, a large variety of grains can be formed. These grains can be of different color, size, and consistency, depending on the causative micro-organism [2]. The grains of the most common causative agent *M*. *mycetomatis* are black, firm, and brittle and are 0.5–1 mm in size [3]. They consist of densely packed fungal mycelia embedded in a hard and brown-black cement material. The chemical composition of these grains is not fully understood, but lipids, proteins, DHN-melanin, Cu, Zn and Ca are known to be present in the grain [4–6]. Surrounding the grain, an extensive granuloma formation is present, characterized by a large zone of neutrophils.

Interestingly, although grain formation is key in mycetoma, its formation process is not well understood. This is because grain synthesis cannot be induced *in vitro*, a mammalian host is necessary [2,7,8]. In the past, mice and monkeys were used to induce grain formation and mimic *M*. *mycetomatis* mycetoma [7,9–12], with various success rates. Histologically, the grains induced in mammal models indeed resemble the grains formed inside the human body, characterized by mycelia embedded in cement material and a neutrophil zone surrounding them.

Since in both human patients and in infected animals neutrophils were found surrounding the mycetoma grain, these cell types might be important in the formation of the mycetoma grain. These cells, however, are probably not the sole factor involved, since exposure of *M*. *mycetomatis* towards neutrophils did not induce grain formation *in vitro* (van de Sande, personal communication). Therefore a non-mammalian host in which neutrophil-like cells are present might be a suitable alternative to induce grain formation. One such host, is the larvae of the greater wax moth *Galleria mellonella* [13–19]. These larvae have an immune system with similarities to the mammal innate immune response [20]. The *G*. *mellonella* immune response consists of two tightly interconnected components: the cellular and the humoral responses [20]. The cellular response is mediated by hemocytes and involves responses such as phagocytosis [20]. The humoral defense is characterized by anti-microbial peptides, complement-like proteins such as peroxynectin, transferrin, lysozyme and defensin [20]. The process of phagocytosis, the production of reactive oxygen species and degranulation is similar between hemocytes and neutrophils [20]. Also receptors such as Toll like receptors and beta glucan receptors, and the formation of a neutrophil extracellular net are similar between hemocytes and neutrophils [20].

Until now, invertebrate hosts were never used to study the pathogenesis of mycetoma, but they might be appealing alternatives to the mammalian hosts since they are inexpensive to keep, easy to manipulate, and they can be kept at 37°C, which makes the comparison of pathogenic processes inside the human body possible [19–21]. Therefore, in the present study, we investigated if *G*. *mellonella* larvae can serve as an alternative model host to study the grain formation in *M*. *mycetomatis*. We evaluated the survival of larvae when infected with different *M*. *mycetomatis* isolates and we verified the presence of grains in the tissue by histopathology. Our results demonstrate that larvae of *G*. *mellonella* can be used as an alternative to mice to study grain formation in *M*. *mycetomatis* mycetoma.

## Materials and Methods

### *G*. *mellonella* larvae

Final sixth instar *G*. *mellonella* larvae were acquired from Vellinga Voedseldieren, Ridderkerk, The Netherlands and kept at room temperature on wood shavings in the dark until use. Larvae were used within 5 days of receipt. Larvae of approximately 300 to 500 mg showing no discoloration were selected for the experiments.

### Fungal strains and growth conditions

**Fig 1:**
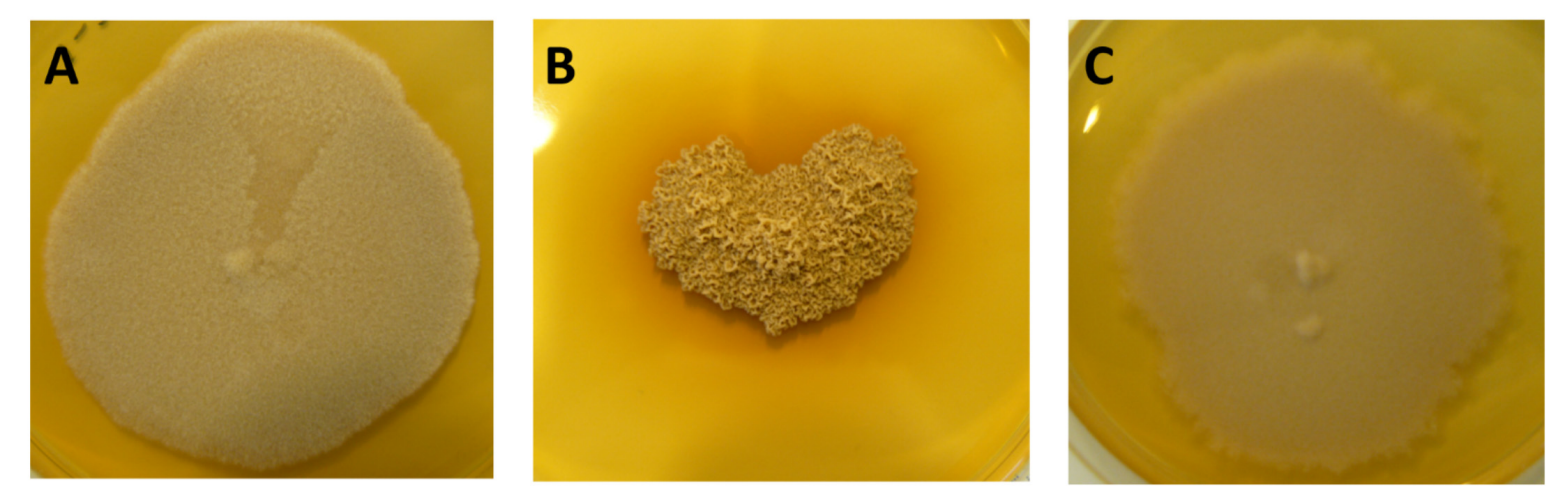
Different morphologies of *Madurella mycetomatis* strains used to infect *Galleria mellonella* larvae. Strains mm55 (panel A), mm68 (panel B) and cn796 (panel C) grown on Sabouraud agar for 4 weeks at 37°C. Strain mm68 clearly has a different morphology than strains mm55 and cn796.

*M*. *mycetomatis* strains mm55, mm68 and cn796 were selected for this study based on their genetic differences (different AFLP types) and different morphology (Fig 1) [22]. These isolates were isolated by direct culture of the black grains obtained by deep surgical biopsies from three different mycetoma patients seen in the Mycetoma Research Centre in Sudan. The strains were identified to the species level by morphology, polymerase chain reaction with *M*. *mycetomatis* specific primers and sequencing of the internal transcribed spacer [23]. The isolates were maintained in the laboratory on Sabouraud agar (Difco laboratories, Becton and Dickinson, Sparks, USA). To prepare the inoculum for the *G*. *mellonella* larvae, *M*. *mycetomatis* mycelia were cut from agarplates and transferred to 200 ml colorless RPMI 1640 medium supplemented with L-glutamin (0.3 g/liter), 20 mM mopholinepropanesulfonic acid (MOPS) and chloramphenicol (100 mg/liter; Oxoid, Basingstroke, United Kingdom). The mycelia were disrupted by 20 s sonication at 28 micron (Soniprep, Beun de Ronde, The Netherlands) and incubated for 2 weeks at 37°C. To prepare the inocula, mycelia were separated and washed by vacuum filtration (Nalgene, Abcoude, The Netherlands). Wet weights of the mycelia were determined and a suspension containing 100 mg wet weight per ml in phosphate-buffered saline (PBS) was sonicated for 2 min at 28 micron. This procedure does not kill the fungus, and results in a homogenous hyphal suspension [7]. The resulting homogenous suspension was washed once in PBS and further diluted to their final inoculum size. To confirm fungal viability and to exclude bacterial contamination, 10 μl of each suspension was cultured on blood agar and Sabouraud agar (Difco Laboratories, Becton Dickinson) for 2 weeks at 37°C.

### Infection of *G*. *mellonella* larvae

*G*. *mellonella* larvae were inoculated with various amounts of viable *M*. *mycetomatis*, ranging from 0.04 to 4 mg wet weight per larvae. Inoculation was performed by injecting 40 μl of the fungal suspension in the last left pro-leg with an insulin 29G U-100 needle (BD diagnostics, Sparsk, USA). As controls, untouched larvae, larvae pricked with the needle and larvae injected with PBS were included. For the survival experiments, each group consisted of 15 larvae. To determine the burden of infection, each group consisted of 5 larvae. Larvae were kept at 37°C during the experiment.

### Survival curves

To monitor the course of infection, larvae were checked daily for survival for 10 days. If during these 10 days larvae formed pupa, these individuals were left out of the equation, since we could not ascertain that these individual larvae would have died during the infection or would have survived. At 4h, 24h, day 3, day 7 and day 10 after inoculation larvae were dissected to determine if black grains were present by macroscopic observation and histology. If grains were present, culture on Sabouraud agar was performed to determine their viability.

### Histology

For histological observations, larvae were fixed in 10% buffered formalin. Since the larval exoskeleton is impenetrable to most fixative reagents, 100 μl of the 10% buffered formalin was injected into the larvae [24]. After 24 h fixation, whole larvae were dissected longitudinally into two halves with a scalpel and fixated in 10% formalin for at least another 48 h [24]. The two halves of larvae were routinely processed for histology. Sections were stained with hematoxylin and eosin (HE), Grocott methanamine silver and Sirius red. To compare the grains formed in the *Galleria mellonella* larvae, histological slides were obtained from 3 human patients (Mycetoma Research Centre) and the mouse model developed by Ahmed et al [7]. One of the strains used to infect our *G*. *mellonella* larvae (strain mm55) was previously used by Ahmed et al. for developing the mice model [7].

### Fungal burden

To determine the fungal burden 5 larvae from each group were sacrificed at 4h, 24h, 3 days, 7 days or 10 days post infection. To each larvae 10 chrome steal metal balls and 1 ml PBS were added. Larvae were homogenized in a Qiagen Tissue lyser for 5 min at 30 Hz. From each homogenized larvae 250 μl undiluted, 50 μl undiluted and 50 μl 1:10 diluted suspension was plated out on Sabouraud-gentamicin agar. Plates were incubated at 37°C for two weeks and the number of colony forming units per larvae (CFUs) was determined.

### Quantification of melanization

To quantify the melanization, haemolymph of each larvae was collected 4h, 24h, 3 days, 7 days or 10 days post infection as described by others [25]. In short, the haemolymph was harvested by making a small incision below the last proleg of the larvae. The haemolymph was collected into clean 1.5 ml tubes and diluted 1:10 with IPS buffer (Insect Physiological Saline: 150 mM sodium chloride, 5 mM potassium chloride, 10 mM Tris-HCl pH 6.9, 10 mM EDTA and 30 mM sodium citrate) immediately after collection. The optical density of the diluted heamolymph was measured at 405 nm to determine melanisation as described by others [24]. Each hemolymph was measured three times independently.

### Statistical analysis

To compare the survival curves the Log-rank test was performed. To compare differences in CFU count and melanization between the different groups, the Mann-Whitney test was performed. A p-value smaller than 0.05 was deemed significant.

### Ethical statement

For the isolated strains and histological sections used from patients, written informed consent was obtained from all participants and ethical approval was obtained from Soba University Hospital Ethical Committee, Khartoum, Sudan. The histological sections used from mice were obtained from a previous study performed by Ahmed et al. [7]. For that study, approval was obtained from the Animal Care and Use Committee of the Erasmus Medical Center, Rotterdam, The Netherlands. The experimental protocols adhered to the rules specified in the Dutch Animal Experimentation Act of 1977 and the published Guidelines on the Protection of Experimental Animals by the Council of the European Community of 1986.

## Results

### Survival of *G*. *mellonella* larvae after infection with *M*. *mycetomatis*

**Fig 2:**
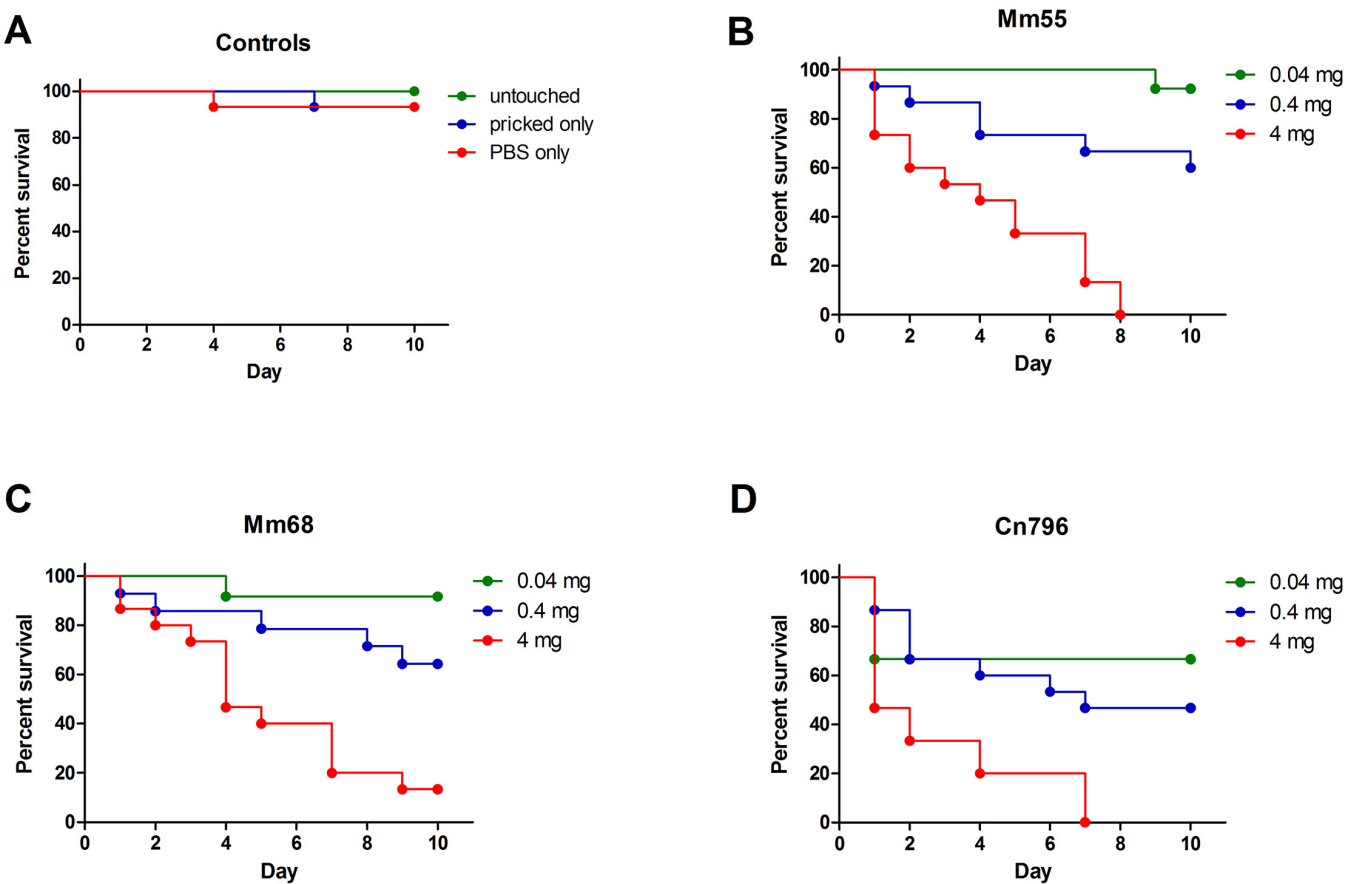
Survival of *Galleria mellonella* larvae infected with *Madurella mycetomatis*. In panel A, the survival curves of the control larvae are shown. For each experiment three different controls were used: untouched larvae, larvae only pricked by the needle and larvae injected with PBS. As is seen in this panel, high survival was obtained for all control groups. In panel B, the survival of larvae infected with 0.04, 0.4 and 4 mg mm55 are shown. More than 90% of the larvae survived when they were infected with only 0.04 mg mm55, no larvae survived when they were infected with 4 mg mm55. In panel C, the results for strain mm68 are shown. As is seen in this graph, when larvae were infected with 4 mg mm68, still some survival is noted. In panel D, the survival of larvae infected with 0.04, 0.4 and 4 mg cn796 are shown. Again at the highest inoculum all larvae died.

To establish a grain model in *G*. *mellonella* larvae, three different *M*. *mycetomatis* isolates were selected. These isolates differed in morphology (Fig 1) and genetic make-up [22]. As can be seen in Fig 2, a concentration-dependent survival was noted when *G*. *mellonella* larvae were infected with *M*. *mycetomatis* strains mm55, mm68 and cn796. For mm55 and cn796 an inoculum of 4 mg wet weight per larvae resulted in death in all *G*. *mellonella* larvae within 7 and 8 days, respectively. This inoculum resulted in significant decreased survival compared to the PBS controls (Log-rank, p<0.0001 for all strains), the 0.04 mg inoculum (Log-rank, p<0.001 for strains mm55 and mm68 and p = 0.0009 for strain cn796) and the 0.4 mg inoculum (Log-rank, p = 0.0003 for mm55, p = 0.004 for mm68 and p = 0.003 for cn796). An inoculum of 0.04 mg resulted in a 90% survival in the case of mm55 and only a 70% survival in the case of cn796. The survival obtained when this inoculum was used, did not differ significantly from the survival obtained in the PBS injected controls (Log-rank, p>0.05 for all strains).

**Fig 3:**
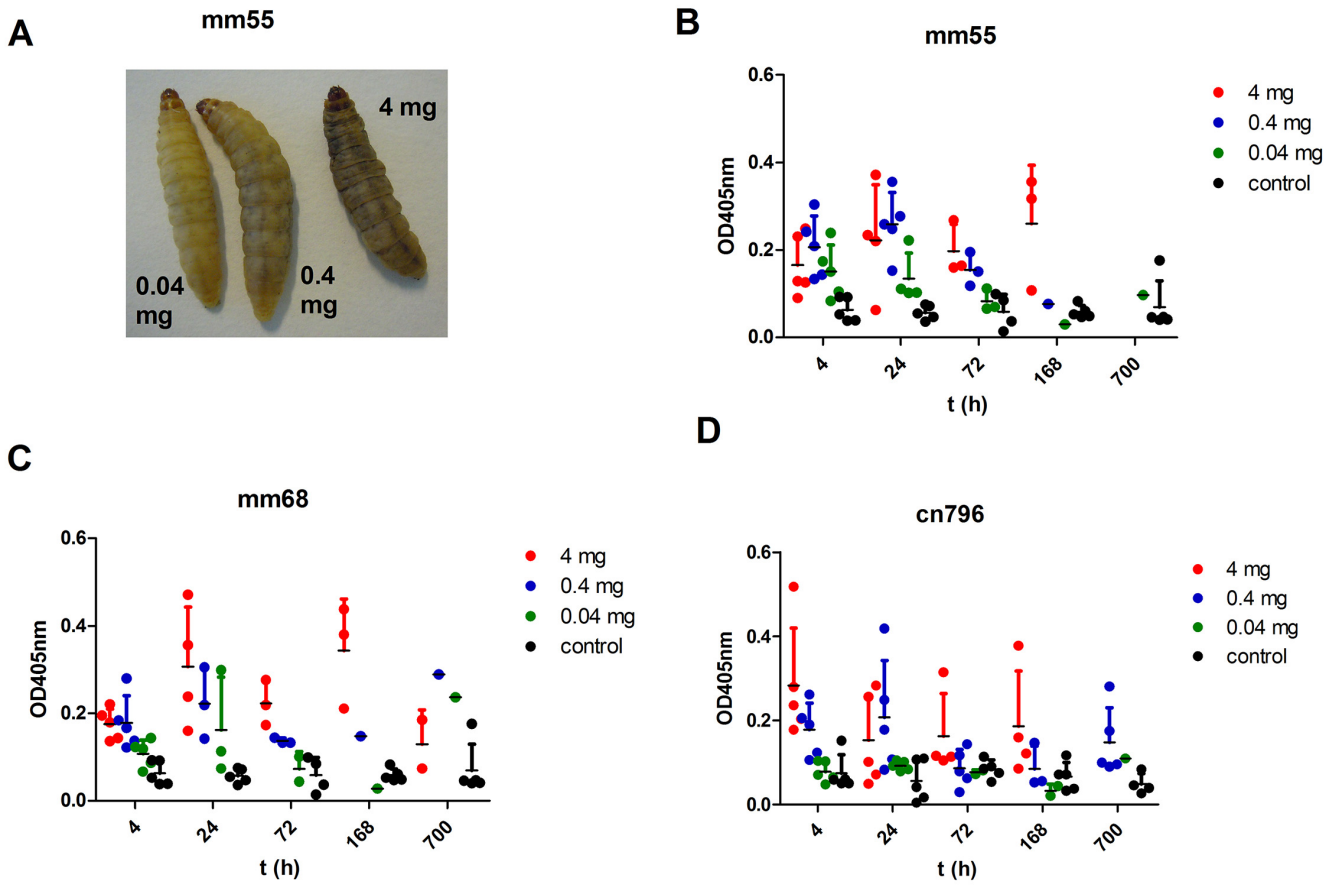
Melanization of *Galleria mellonella* larvae during *M*. *mycetomatis* infection. In panel A, three larvae infected with 0.04, 0.4 and 4 mg mm55 are shown. As is seen, the larvae infected with 0.04 mg mm55 is not melanised (left larvae), the larvae infected with 0.4 mg mm55 is slightly melanised (middle larvae) and the larvae infected with 4 mg mm55 is heavily melanised (right larvae). In panels B, C and D, the melanization of the haemolymph is demonstrated by measuring the OD_405nm_ of the haemolymph at time points; 4h, 24h, 72h, 168h and 700h after infection. In panel B, the melanization of larvae infected with mm55 is shown, in panel C those infected with mm68 and in panel D those infected with cn796. At all time-points it appeared that the larvae infected with the highest inoculum of *M*. *mycetomatis* were the most melanised, those with the lowest inoculum, were the least melanised.

Since melanization is a key step in the antimicrobial response of *G*. *mellonella* upon infection, the pigmentation of the larvae after *M*. *mycetomatis* challenge was assessed both by observation and by measuring the melanization in the haemolymph (Fig 3). Larvae infected with 0.04 mg wet weight of mm55, mm68 or cn796 showed no obvious melanization during the 10 days observation period. They looked similar to the non-infected larvae. When the haemolymph itself was assessed for melanization, a slight elevation was noted when compared to the uninfected controls, but this elevation was not significant (Mann Whitney, p>0.05) (Fig 3B and 3C and 3D). Larvae infected with 0.4 mg wet weight mm55, mm68 and cn796 showed light melanization (Fig 3A). Melanization occurred within 4 hours after inoculation of the fungi. As is seen in Fig 3B–3D, the haemolymph itself was significantly darker when compared to the uninfected controls (Mann Whitney, p = 0.016 for mm55 and p = 0.008 for mm68 and cn796). When larvae were infected with 4 mg wet weight of either one of the strains, strong melanization was noted within 4 hours after inoculation (Fig 3A). The haemolymph of the surviving larvae was again significantly darker when compared to the uninfected controls (Mann Whitney, p = 0.016 for mm55 and cn796 and p = 0.008 for mm68). When melanization was present, this remained present until the end of the observation period (day 10).

### Grain formation during the infection

**Fig 4:**
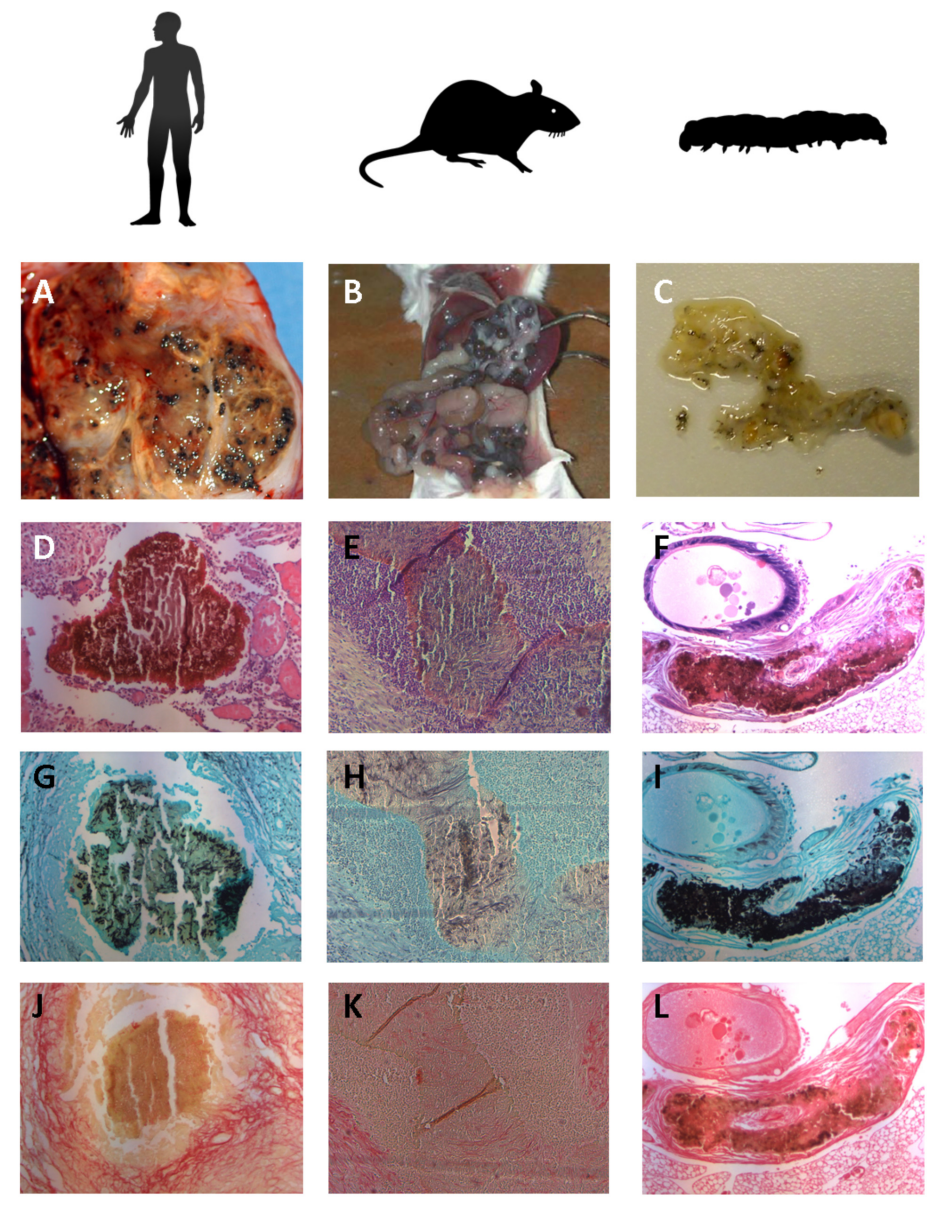
Grain formation in human, mouse and larvae of *Galleria mellonella*. In panels A, B and C the presence of grains in tissue is shown. In panel A, black grains are clearly visible inside an excised part of the mycetoma lesion of a human patient. In panel B, grain formation inside a Balb/c mouse is shown. In panel C, grains inside a dissected *G*. *mellonella* larvae are demonstrated. In panel D, E and F, 100 times magnified HE slides of mycetoma grains show that the grains are surrounded with immune cells, and a large zone of neutrophils is present in both human and mice. In *G*. *mellonella* larvae these immune cells are not present yet (panel F) at a time point of 3 days after infection. In grains obtained from human, mouse and *G*. *mellonella* larvae fungal hyphae can be seen as demonstrated with Grocott staining (panels G, H, I). With Sirius Red staining a clear encapsulation of grains with collagen is seen in human, mouse and *G*. *mellonella* larvae (panels J, K, L).

**Fig 5:**
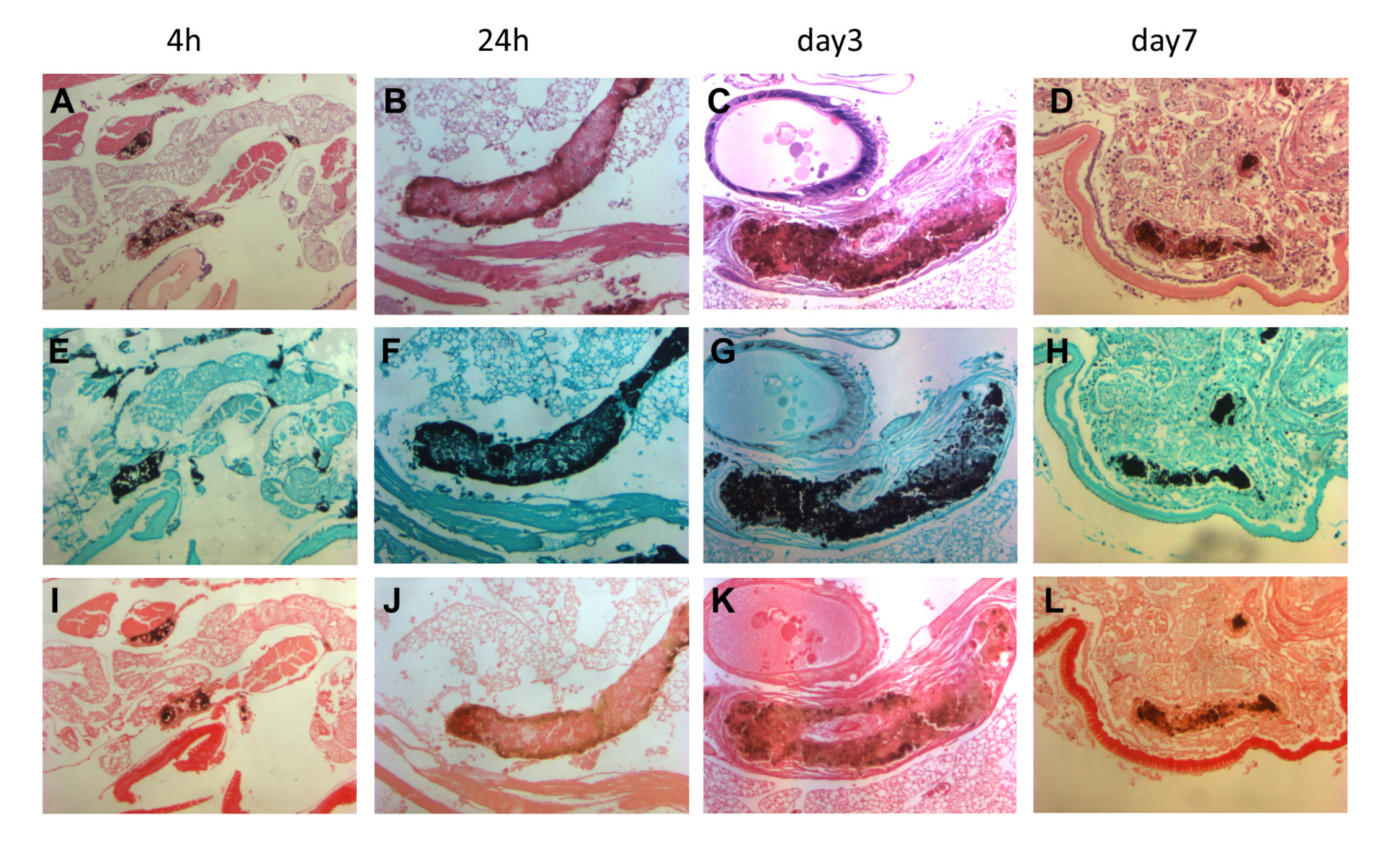
Grain formation over time. Grain formation of *M*. *mycetomatis* strain mm55 within *Galleria mellonella* larvae (100 times magnified). At 4h, 24h, 3 days and 7 days post infection grain formation is shown. Histological slides are stained with HE (panels A,B,C,D), Grocott (E,F,G,H) to demonstrate the presence of fungal hyphae and Sirius red to demonstrate the presence of collagen(I, J, K, L). As is seen in panels A, E and I small grains are already visible at 4h after infection. Hardly any immune cells are present (panel A), and no collagen is seen surrounding the grain (panel I). After 24h the grains are larger and cement material starts to form (panel B). At 3 days post infection, the grain has become encapsulated with collagen (panel K). This collagen capsule disappears at day 7 (panel L), a time point at which many immune cells are shown to be present surrounding the grain (panel D).

A key feature of mycetoma is the formation of grains. To observe if grains were formed during the infection, larvae were dissected 4h, 24h, 3 days, 7 days and 10 days after infection. As can be seen in Table 1, even at the lowest inoculum, black spots were seen after 4h of infection. The lowest inoculum resulted in only a few black spots, while the highest inoculum resulted in numerous spots. To determine if the black spots indeed represented the characteristic mycetoma grains, histological slides were prepared and stained with HE, Grocott and Sirius red. These were compared to histological slides from patients and from mice (Fig 4). As is seen in Fig 5, even after 4 hours grains were visible. At this time point grains were still forming, and although cement material was already present, some immune cells were seen within the grain. From day 3 onwards, these immune cells disappeared from within the grain, and the cement material was found throughout the grain. Characteristic of human and mouse grains is the presence of a collagen capsule surrounding the grain. No collagen capsule was present at 4h and 24h after inoculation. On day 3, larvae infected with either mm55 or mm68 had formed collagen capsules (Figs 4 and 5). These capsules disappeared later in infection (day 7 for mm55 and day 10 for mm68). No capsule was found for Cn796. Surprisingly, at the same time when the capsule disappeared, an increase in the presence of immune cells was noted.

**Table 1:** Grain formation during infection.

	Grain formation in surviving larvae				
	4h	24h	3 days	7 days	10 days
Mm55					
0.04 mg/larvae	5/5	5/5	5/5	5/5	5/5
0.4 mg/larvae	5/5	5/5	5/5	5/5	5/5
4 mg/larvae	5/5	5/5	2/2	2/2	No survival
Mm68					
0.04 mg/larvae	5/5	5/5	5/5	4/5	5/5
0.4 mg/larvae	5/5	5/5	4/4	5/5	5/5
4 mg/larvae	5/5	5/5	2/2	2/2	2/2
Cn796					
0.04 mg/larvae	5/5	5/5	4/4	5/5	5/5
0.4 mg/larvae	5/5	5/5	5/5	3/3	5/5
4 mg/larvae	5/5	2/2	3/3	No survival	No survival

**Fig 6:**
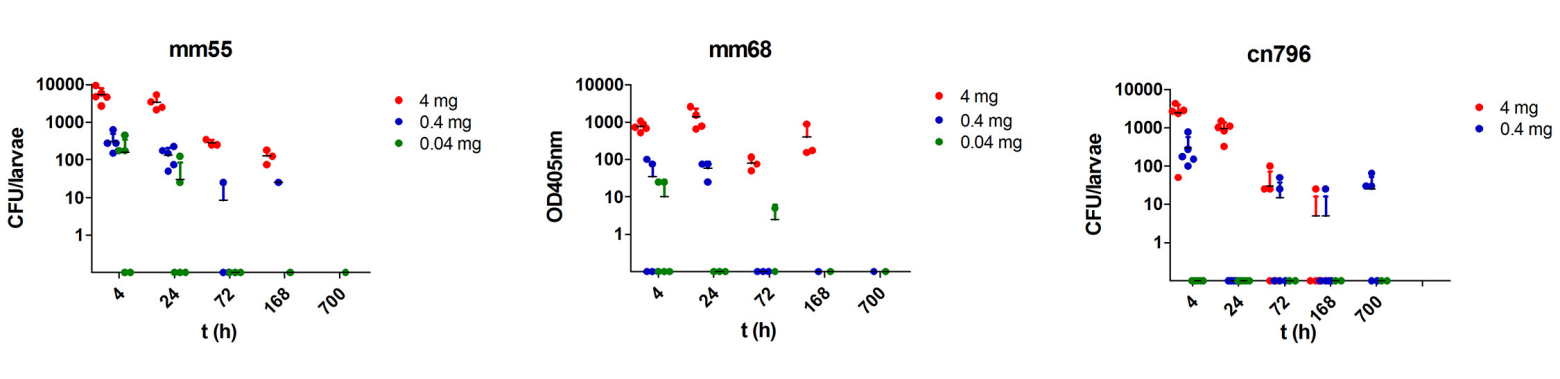
Assessment of fungal burden over time. In panels A, B and C, the number of colony forming units per larvae are shown. At time points 4h, 24h, 72h, 168h and 700h after infection larvae were sacrificed and homogenized. For each larvae, 250 μl undiluted, 50 μl undiluted and 50 μl 1:10 diluted suspension was plated out on Sabouraud-gentamicin agar. In panel A, the fungal burden of larvae infected with 4 mg/larvae (red), 0.4 mg/larvae (blue) or 0.04 mg/larvae (green) mm55 is shown. In panels B and C, the same is demonstrated for strains mm68 and cn796. At all time-points it appeared that the larvae infected with the highest inoculum of *M*. *mycetomatis* had the highest concentration of *M*. *mycetomatis* in their haemolymph. For the lowest concentration, the CFU dropped below the detection limit as early as 3 days after infection.

The fungal burden inside the larvae was assessed by culturing homogenate of sacrificed larvae. After one week of culturing on sabouraud agar, clear colonies were grown from grains originating from strains mm55 and cn796, two weeks were necessary to discriminate the colonies of mm68. Interestingly, the morphology of the colony originating from the grains was similar to the colony morphology of the isolate before passage through the larvae. As is seen in Fig 6, the number of CFU was dependent on the starting inoculum. Significant higher CFUs were obtained for the 4 mg inoculum at time points 4h and 24h as for the 0.4 and 0.04 mg inoculum for all strains (Mann-Whitney, p = 0.08 for all strains at 4h and p = 0.016 for mm55, p = 0.05 for mm68 and p = 0.08 for cn796 at 24h). After 72h the number of CFUs dropped considerably, the initial differences in number of CFUs observed for the different inoculum sizes was no longer observed (Mann-Whitney p>0.05, for all strains at time points 72h, 168h and 700h). The number of CFU obtained from the homogenate of the sacrificed larvae was the highest at 4h and 24h after inoculation, the load lowered during the infection. The initial difference in CFU was comparable at 4h and 24h after infection for all strains (Mann-Whitney, p>0.05), but after 72h the number of CFU dropped considerably (4 mg inoculum, Mann-Whitney, p = 0.04 for strains mm55 and mm68, and p = 0.01 for strain cn796), Although at some time-points the load dropped below the detection limit of the method used, if individual grains were isolated and cultured from the infected larvae, *M*. *mycetomatis* could still be retrieved, even from larvae infected with the lowest inoculum.

### Differences in virulence between the three *M*. *mycetomatis* isolates

To determine if the three morphologically different *M*. *mycetomatis* isolates (Fig 1) also differed in pathogenicity in *G*. *mellonella* larvae, we compared survival rates, melanisation of the infected larvae and the CFU obtained from infected larvae between the different isolates. For this we used a 4 mg inoculum for each *M*. *mycetomatis* isolate. In terms of survival, it appeared that the survival curves obtained for strain mm55 were comparable to those obtained for mm68 (Log-rank, p = 0.24) and cn796 (Log-rank, p = 0.13). Only when strains mm68 and cn796 were compared a significant difference in survival was noted (Log-rank, p = 0.027), cn796 appeared to be more pathogenic to *G*. *mellonella* larvae than mm68. In terms of melanisation, no significant difference in melanisation of the infected larvae was observed for each of the time points monitored (Mann-Whitney, p>0.05). In terms of CFU, initially significantly more CFUs were retrieved for strain mm55 when compared to mm68 (t = 4h, Mann-Whitney, p = 0.008) but this difference was not observed at the later time points (Mann-Whitney, p>0.05). Also compared to cn796, a higher CFU count was observed for mm55. Significantly more CFUs were obtained for mm55 than for cn796 at 24h (Mann-Whitney, p = 0.016), at 72h (Mann-Whitney, p = 0.034) and at 168h (Mann-Whitney, p = 0.026). No difference was observed when the number of CFU counts was compared between mm68 and cn796 (Mann-Whitney, p>0.05).

## Discussion

Mycetoma is a chronic granulomatous infection caused by either bacteria or fungi and characterized by the formation of grains [2]. Worldwide, the fungus *Madurella mycetomatis* is the most common causative agent of mycetoma [1]. Although the disease has been known for more than 200 years, still there are many factors of this disease which are not well understood, including the underlying host factors, the infection route, the process of grain formation and the therapeutic efficacy of the various drugs towards the causative agent [26]. Also simple diagnostic tools are currently lacking, and identification of the causative agent still relies on histology and culturing of the grain, which takes long and misidentifications are common [27]. In order to gain more insight in the pathogenesis of mycetoma and to develop rapid point of care diagnostic tools and effective therapies, a grain model is needed.

Here we demonstrated that *M*. *mycetomatis* grains could be produced in the invertebrate host *G*. *mellonella* which resembled the grains formed in human patients and the mouse model developed by Ahmed et al. [7]. Histologically, the grains obtained in *G*. *mellonella* larvae resembled those obtained in mice and human, only the immune reaction surrounding the grain differed. In mice, a large neutrophil zone surrounding the grain was noted, even when the grain itself was encapsulated with collagen. In *G*. *mellonella* larvae, invasion of immune cells only started when the collagen capsule disappeared. The grain itself looked similar in all three hosts. Not surprisingly, the tissue reaction surrounding the grain was more similar between mice and human than between *G*. *mellonella* and human.

Next to comparing the histology of the grains between the different hosts, our *G*. *mellonella* model can also be compared with the mouse model of Ahmed et al. in terms of pathogenicity of the fungus, the survival and the efficacy of grain formation. In terms of the pathogenicity of the fungus, in both models the *M*. *mycetomatis* inoculum was prepared in a similar manner by sonication. Furthermore, in both models *M*. *mycetomatis* strain mm55 was used. Ahmed et al, used inocula ranging from 0.8 mg to 120 mg per mouse, corresponding to approximately 0.04 mg to 8 mg *M*. *mycetomatis* per gram mouse [7]. We used a similar inoculum range in our *G*. *mellonella* model, namely 0.08 mg *M*. *mycetomatis* per gram larvae (0.04 mg/larvae) to 8 mg/g (4 mg/larvae). In the mouse, complete survival was found at inocula up to 4 mg/g after 38 days, while only 37,5% of mice survived at an inoculum of 8 mg/g [7]. In terms of survival, the *G*. *mellonella* host appeared to be slightly more susceptible towards a *M*. *mycetomatis* infection, since even at concentrations as low as 0.08 mg/g, not all larvae survived. At an inoculum of 8 mg/g, no larval survival was noted. In terms of grain formation, the *G*. *mellonella* model appeared to be more efficient. In mice, an adjuvant was needed to induce grain formation, while in *G*. *mellonella* larvae no adjuvant was needed at all [7]. Furthermore, even at the lowest inoculum tested (0.04 mg/larvae (0.08 mg/g), grains were formed in all larvae. In mice, at such low concentrations no grains were obtained. Only at a concentration of 8 mg/g grains were formed in all inoculated mice [7].

Histologically, the *M*. *mycetomatis* grains formed in the *G*. *mellonella* larvae looked similar to those formed in mice or human, which could make this model a valuable model to further investigate the process of grain formation, to develop novel diagnostic tools or to determine the efficacy of antifungal agents against this protective structure.

In order to investigate the process of grain formation, the grain model described here can be used to determine the pathogenicity of genetically modified *M*. *mycetomatis* isolates. Here we already showed that grains can be formed when different *M*. *mycetomatis* isolates are used and that small differences in terms of survival between these isolates were observed. By generating genetically modified *M*. *mycetomatis* isolates, and using them to infect *G*. *mellonella* larvae it can be determined which genes are essential in the grain formation process. For several other fungal infectious diseases the pathogenicity of genetic mutants have been studied in *G*. *mellonella* larvae [13,18]. Studying the pathogenicity of these genetic mutants in *G*. *mellonella* is only useful if they could predict that the results obtained are similar as in the patient. For other fungal infections it has been determined if various genetically modified isolates resulted in either enhanced or reduced pathogenicity in different hosts including insects and mammals. By studying the pathogenicity of *Aspergillus fumigatus* mutants in the siderophore and folate biosynthesis pathways in different hosts, it could be concluded that similar survival rates were obtained in G. *mellonella* larvae and mice [18]. In contrast, when the pathogenicity of these mutants were assessed in another invertebrate model, namely *Drosophila melanogaster*, no comparable results were obtained [18], demonstrating that not all invertebrate hosts are able to mimic the outcomes of mammalian hosts. Also, 9 out of 10 different *Candida albicans* mutants which were tested for virulence in both *G*. *mellonella* larvae and mice, showed similar pathogenicity. Only the cph1efg1 double mutant of *C*. *albicans* differed, appeared to be virulent in the larvae and avirulent in mice [13]. Since for other fungal species similar results were obtained when the pathogenicity of genetically modified isolates were tested in *G*. *mellonella* larvae and in mice, it is possible that this approach can also be used in the future to determine which *M*. *mycetomatis* genes are essential for grain formation.

Another way in which this *G*. *mellonella* grain model can be exploited in the future is to use it as source of fresh grains which can be used in the development of rapid point-of-care diagnostic tools. It will allow to develop antigen-based diagnostic tools and DNA-based diagnostic tools directly from the grain, which was not possible in the past. Grain specific antigens could be detected and novel methods to isolate DNA directly from patient material could be developed. This would make the culture step redundant for molecular diagnostic tools and would shorten the time to identification with more than 6 weeks.

May be the most important use of this grain model in the near future is to study the effect of different antifungal agents against *M*. *mycetomatis* grains. Currently, only *in vitro* models have been used to determine the susceptibility of *M*. *mycetomatis* towards the different antifungal agents [28,29]. In these *in vitro* susceptibility assays, hyphal fragments were generated in RPMI medium and exposed to different concentrations of antifungal agents. It appeared that *M*. *mycetomatis* itself was highly susceptible towards azole antifungal agents, but it is difficult to translate these models directly to the patient situation since no grains are formed. The only study, which ever compared the susceptibility of *in vitro* grown *M*. *mycetomatis* isolates and grains already demonstrated that antifungal agents penetrate a hyphae better than a grain [30]. Furthermore, recently it was also demonstrated that although itraconazole has a better *in vitro* activity against *M*. *mycetomatis* than amphotericin B, it could not prevent grain formation in a mouse model, while amphotericin B could [31]. This indicates that there is indeed a difference between the activity of antifungal agents against *M*. *mycetomatis* hyphae *in vitro* and *M*. *myctomatis* grains *in vivo*. Therefore the infected *G*. *mellonella* larvae can be used to study the *in vivo* efficacy of the different antifungal agents against *M*. *mycetomatis* grains.

In conclusion, in this study we show that the larvae of the greater wax moth *G*. *mellonella* can be used to induce grain formation *in vivo* and that the grains produced resemble the grains found in experimental mouse models and in human. The *G*. *mellonella* grain model could serve as a useful model to study the grain formation and therapeutic response in the future.
